# Perspectives of Medical Students and Developers Regarding Virtual Reality, Augmented Reality, Mixed Reality, and 3D Printing Technologies: Survey Study

**DOI:** 10.2196/54230

**Published:** 2024-05-07

**Authors:** Young Hyun Yun, Dong Hoon Shin, Hyung Jin Choi

**Affiliations:** 1 Department of Anatomy and Cell Biology Seoul National University College of Medicine Seoul Republic of Korea; 2 Department of Biomedical Sciences Seoul National University College of Medicine Seoul Republic of Korea

**Keywords:** medical student, developer, virtual reality, augmented reality, mixed reality, 3D printing, perspective, survey

## Abstract

**Background:**

Emerging technologies, such as virtual reality (VR), augmented reality (AR), mixed reality (MR), and 3D printing (3DP), have transformative potential in education and health care. However, complete integration has not yet been achieved, and routine use is limited. There may exist gaps in the perspectives of these technologies between users and developers, and improvement may be necessary in developing such technologies.

**Objective:**

The purpose of this study was to investigate the gaps in perspectives between medical students and developers in medical education regarding satisfaction and anticipated future use of VR, AR, MR, and 3DP technologies, as well as developers’ perspectives on their advantages and current challenges.

**Methods:**

This retrospective survey study was conducted during a 4-hour elective course over a period of 4 weeks. In this course, computed tomography scans of congenital heart disease patients, medical image processing software, head-mounted displays, and a virtual table were used. Student pre- and postsurveys and the developer survey included demographic and other characteristics, satisfaction, and anticipated future use of VR, AR, MR, and 3DP technologies. The advantages and current challenges of these technologies were only assessed in the developer survey.

**Results:**

The study enrolled 41 participants, including 15 first-year medical students and 26 software and content developers. Students were more satisfied than developers across AR, VR, and 3DP in terms of overall satisfaction (VR and AR: *P*<.001; 3DP: *P*=.002), esthetics (VR: all *P*<.001; AR: vividness, *P*=.006 and design, *P*<.001; 3DP: vividness, *P*=.001 and design, *P*=.002), and continuous use intention (VR: repetition, *P*=.04 and continuous use, *P*=.02). Particularly in VR, satisfaction with reality was higher among students than among developers (real world, *P*=.006). Developers anticipated future use of MR for educating medical students and residents, individual and collaborative surgical planning, and performing surgery on patients. In contrast, students anticipated future use of VR primarily for student education, 3DP for resident education and individual surgical planning, and AR for collaborative surgical planning and performing surgery on patients. Developers perceived the inherent capabilities of VR, AR, and MR technologies as strengths, with hardware performance identified as a drawback. For 3DP, the possibility of customized product manufacturing was seen as an advantage, while cost was seen as a disadvantage.

**Conclusions:**

This study elucidated the different perspectives between medical students and developers regarding 3D technologies, highlighting the discrepancy in potential applications and challenges within the medical field. These findings will guide the integration of 3D technologies in education and health care to fulfill the needs and goals of both medical students and developers.

## Introduction

### Background

In recent years, 3D technologies, including virtual reality (VR), augmented reality (AR), mixed reality (MR), and 3D printing (3DP), have shown considerable potential to revolutionize the fields of education and health care [1-3]. Each of these technologies offers different capabilities. The basic principle of VR involves the immersion of users in artificial environments, providing complete immersion and removing them from their immediate surroundings [4]. On the other hand, the principle of AR involves overlaying digital information onto the real world, allowing interaction between the physical and digital realms [5]. MR goes a step further by merging tangible and virtual worlds [6]. Unlike VR, which fully immerses the user in a virtual environment, and AR, which overlays digital information onto the physical world, MR is defined as a technology that seamlessly merges the physical world with the virtual world, allowing physical and digital objects to coexist and interact in real time [7]. In MR, virtual objects appear to exist in the same space as physical objects, and users can interact with both in a natural and intuitive way. These technologies are commonly experienced using headsets, allowing hands-free viewing of digital information within the user’s view. Additionally, 3DP is a method of creating a 3D object layer by layer from a computer-generated design [5]. Beyond these basic principles, these technologies have become powerful tools in facilitating practical training and skill development. Numerous studies have highlighted the importance of integrating these cutting-edge technologies into medical and surgical education [8-10].

VR is versatile with applications in 3D anatomical models, surgical planning, and medical skills practice simulators [11]. It has been actively evaluated for its efficacy in anatomy education and has been often compared with traditional methods like dissection and lectures, as well as modern techniques like 2D images and blended instructions [12,13]. In surgical education, VR can effectively address challenges, such as the shortage of available mentors, optimization of training time, and mitigation of the complexities associated with operative procedures [14]. Additionally, by replicating complex surgical scenarios in a controlled and risk-free environment, VR offers a safe space for trainees to enhance their skills and decision-making processes.

AR has been incorporated into different phases of medical training, and it serves as an essential tool for anatomical instruction, which can assist students during classroom studies, a tool in image-based training simulators, and an interactive platform to improve clinical skills [15]. The integration of AR has revolutionized medical education by providing students with real-time visualizations of complex anatomical structures and creating interactive and immersive learning experiences that deepen their understanding of medical concepts [16]. In addition, AR-based training simulators enable learners to improve their practical skills and confidence by allowing them to practice medical procedures in a simulated digital environment before performing them in clinical settings [17]. There is a key difference between AR-based training simulators and VR-based training simulators. VR-based training simulators simulate the actual workspace within a 3D modeling environment and involve the handling of virtual objects using controllers. However, AR-based training simulators allow users to interact with digital elements while still being aware of their physical surroundings [18]. This allows for a more seamless integration of virtual elements with real-world objects and scenarios, offering unique training opportunities that VR alone may not be able to provide.

MR has rapidly advanced in recent years, establishing itself as a fundamental research direction within the field of intelligent medicine. There are significant numbers of MR applications in surgical training and planning [19,20]. Previous studies have found that by expanding upon conventional computer-assisted surgical techniques, MR offers significant potential for enhancing orthopedic training and needle insertion skills [3,21]. This transformative impact extends beyond the confines of surgical applications, encompassing the sphere of medical education as a whole. Some pioneering research has demonstrated that MR has the potential to enhance the efficacy of conveying intricate content through remote learning, a modality that remains pivotal in the field of education [22].

3DP provides a tangible and immersive approach to medical and surgical education [23]. 3DP enables the production of objects with very intricate details and offers the versatility to print a model with different materials, including hydrogels, thermoplastics and thermosets, metals, and ceramics [2,24]. In addition, personalized patient-specific 3DP models help students understand variation and pathology, while surgical planning benefits from accurate organ replicas that enhance visualization and reduce errors [25]. Trainees train their hands-on skills on 3D-printed models in a risk-free environment, and educators simulate complex cases for better decision-making [26]. Furthermore, 3DP plays a crucial role in advancing medical research by facilitating the prototyping of medical innovations, including devices and implants, thus shaping the future of the field [27,28].

These technologies have been well developed in recent years, and this is reflected in a variety of medical specialties in medical education and health care. While these technologies have been widely used to complement existing methods, they are increasingly becoming integral tools, particularly in settings where conventional approaches face limitations or challenges. In educational settings that have constraints, such as limited access to cadavers, high costs, concerns about formaldehyde exposure, ethical considerations, and challenges posed by pandemics, these technologies have gradually replaced traditional methods for medical students [29,30]. Similarly, in health care, personalized instruments, along with advancements like bone grafting and customized implants, are replacing traditional approaches [31]. As they continue to complement and replace traditional methods, they offer innovative solutions to address various challenges and constraints encountered in both education and health care.

In South Korea, after students are accepted into medical school, they typically spend 2 years in premedical education. During this premedical education, they study basic subjects, such as basic sciences, and other fundamental subjects essential for their medical studies. Therefore, the first year of medical school is an appropriate time to introduce these latest technologies. Because students have already established a solid foundation in basic sciences during their premedical education, they are better equipped to grasp the complexities of these technologies and integrate them into their medical education effectively. The current technology curriculum is situated in the context following the completion of the anatomy course and preceding the commencement of the clinical curriculum. Prior to enrolling in this technology curriculum at Seoul National University College of Medicine, students are exposed to heart content in their human anatomy course using these tools. A survey comparing student evaluation of the same content through traditional education versus the use of these tools revealed that the tools were considerably helpful [32].

### Theoretical Background

While numerous studies have examined the satisfaction and effectiveness among medical students, residents, and fellows [33-36], complete integration into routine education and health care has not been achieved. These studies have primarily focused on users, with little attention given to the perspectives of developers, who are responsible for creating these technologies. Consequently, the findings offer an incomplete picture. As these technologies continue to evolve in medical education, it becomes essential to understand the perspectives of both medical students and developers regarding the technologies.

### Relationship Between Users and Developers

Users and developers are commonly considered 2 distinct groups of people [37]. Due to differences in backgrounds and situations, developers and users often share different and sometimes conflicting interests during the software development process. The root cause of many issues is perceived to be ongoing cultural differences. Other theories suggest that personality differences or even differences in how users and developers think cause these barriers [38]. Developers tend to be achievement-oriented and are intrinsically motivated to develop excellent software, while users are primarily focused on improving efficiency and solving problems [39]. The potential conflict of interest between them can negatively affect the performance of software development. Therefore, a study is needed to understand the gaps in their perspectives regarding the range of tools and techniques, which might support future development. Understanding their perspectives might help in refining the implementation strategies of these technologies in the large scope of curriculum development.

### User and Developer Satisfaction and System Success

User satisfaction is one of the most frequently cited factors for measuring system success and one of the most difficult factors to measure [40]. A great deal of research has been conducted to understand the notion of user satisfaction. User satisfaction, as defined by previous researchers, encompasses meeting user needs [41], positive cognitive responses to system use [42], and measurable effects in projects [43]. In education, satisfaction plays a crucial role as a barrier to continuous use and adoption of these technologies [44]. While people may use various technologies without being fully satisfied with them, in the context of education, satisfaction impacts the effectiveness of learning experiences [45]. Students who are dissatisfied with the technologies used in their education may experience hindered engagement, motivation, and, ultimately, compromised learning outcomes [46]. High satisfaction with technology not only correlates with actual experiences of the technology but also enables individuals to anticipate which technologies may be beneficial in future situations. In addition, considering that developers not only represent the core of the development process but also account for the largest cost in software development, it is necessary to investigate developer satisfaction. Ultimately, developer satisfaction is essential for system development success.

### User and Developer Anticipations Regarding Technology

Anticipations of future use in technological development are more than simply descriptions of future products and systems. These anticipations can change the application process of novel technology in medical education as they guide the actions of technology developers [47]. At the same time, extrapolating future technology from past developments can narrow down the potential paths of technological advancement [48]. However, users play a role in shaping the future of technology, as the shape of technology depends on their anticipations of use [47]. To conclude, it is important to consider anticipations for investigating the gap in medical education. We suggest viewing envisaged sociotechnical futures as negotiation arenas between the present and the imagined futures. There is a lack of knowledge on differences in user and developer anticipations regarding the types of technologies that are likely to be widely used in different scenarios. In medical education, there may remain a gap in the anticipated use of these technologies between medical students and developers, and it may be needed to figure out the differences in anticipations to effectively bridge this gap.

### Conceptual Framework

**Figure 1:**
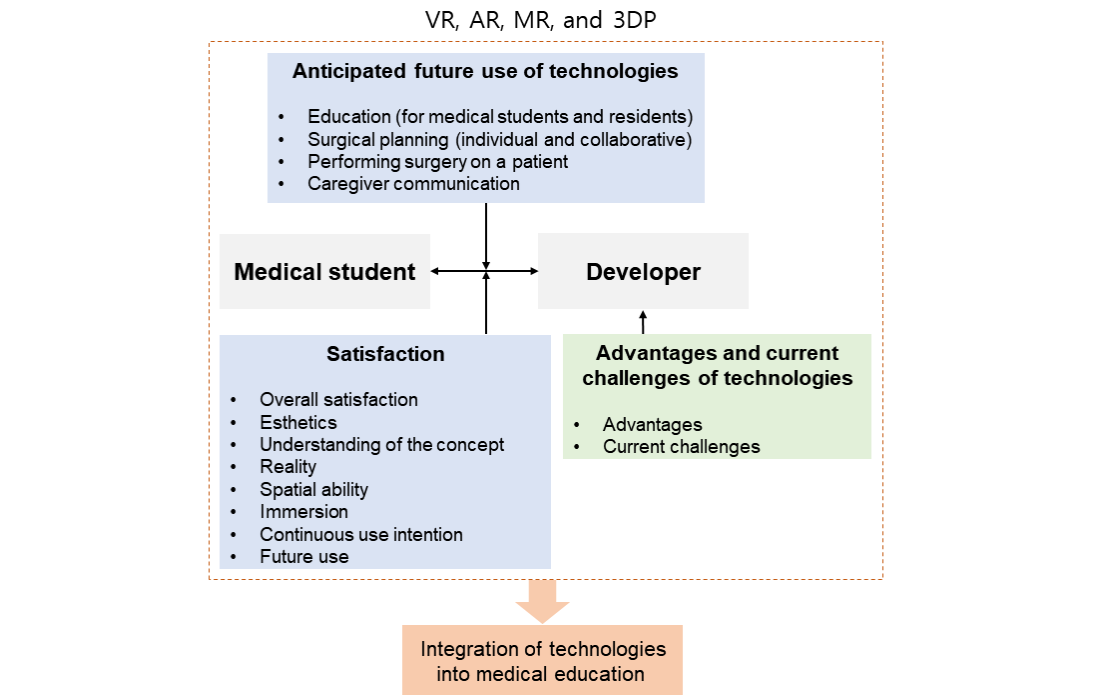
Conceptual framework of this study. 3DP: 3D printing; AR: augmented reality; MR: mixed reality; VR: virtual reality.

This study hypothesized a conceptual framework (Figure 1) in which there is a gap between medical students and developers in terms of satisfaction and anticipated future use of VR, AR, MR, and 3DP technologies, and this gap is associated with the complete integration into medical education. Additionally, from a technological perspective, this study hypothesized that factors related to the advantages and current challenges associated with these technologies from the developers’ perspectives could potentially delay the integration of medical education.

### Study Questions

The aim of this study was to investigate the gap in perspectives between medical students and developers regarding the satisfaction and anticipated future use of VR, AR, MR, and 3DP technologies, as well as developers’ perspectives on their advantages and current challenges. The following specific research questions guided this study:

1. What is the difference in satisfaction levels with VR, AR, MR, and 3DP between medical students and developers?
2. What differences exist in the anticipated future use of VR, AR, MR, and 3DP between medical students and developers?
3. What are the developers’ perceptions of the advantages and current challenges associated with VR, AR, MR, and 3DP?

## Methods

### Participants

All participants voluntarily enrolled in an elective course on 3D imaging software and the applications of 3D technology for human anatomy at Seoul National University College of Medicine, Seoul, Republic of Korea, and were voluntarily recruited. In the academic year 2023, 15 first-year medical students and 26 software and content developers with expertise in VR, AR, or 3DP participated in this study.

### Curriculum and Study Design

The course and the surveys were designed for educational quality improvement purposes prospectively before actual application of the curriculum. The statistical research was performed retrospectively after the completion of the curriculum. 

### Contents and Schedule for the Elective Course

**Figure 2:**
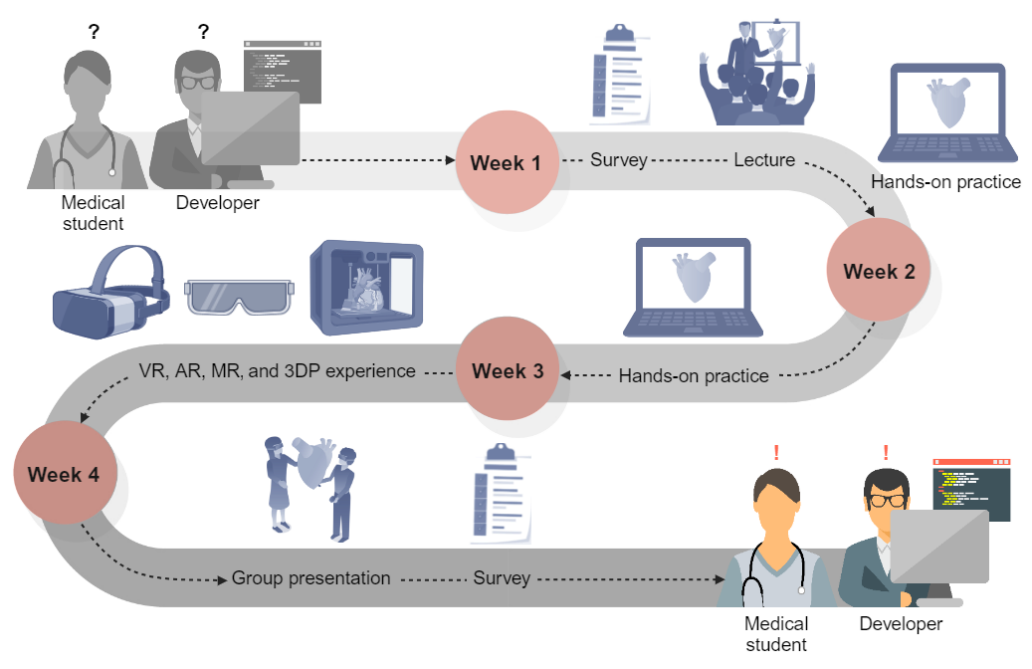
Graphical diagram of the elective course. 3DP: 3D printing; AR: augmented reality; MR: mixed reality; VR: virtual reality.

**Figure 3:**
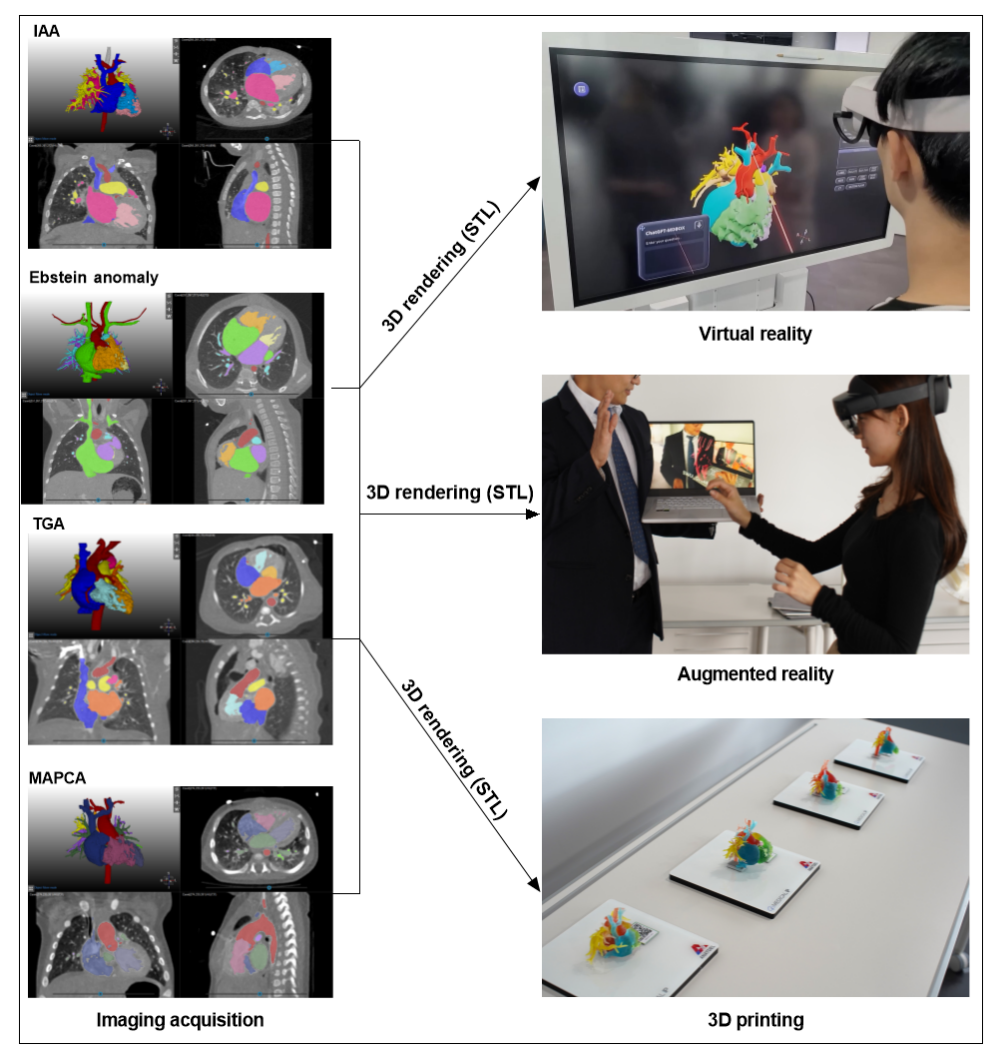
Workflow diagram of hands-on practice for the elective course. Segmented and refined 3D congenital heart disease models are used for not only virtual and augmented reality but also 3D printing. IAA: interrupted aortic arch; MAPCA: major aortopulmonary collateral arteries; STL: standard tessellation language; TGA: transposition of the great arteries.

The elective course spanned 4 weeks with 4 sessions, each lasting 4 hours (Table 1). A graphical diagram of the elective course and a workflow diagram detailing the hands-on practice are shown in Figures 2 and 3. During the initial 3 weeks, the curriculum included lectures and hands-on sessions focusing on the application of artificial intelligence (AI) technology in medical imaging. The AI-based image processing software quickly and automatically segmented the anatomical structures, allowing for adequate processing within the first 3 weeks. Only the finer anatomical structures required separate segmentation. The students were divided into 4 groups and used medical image processing software to outline anatomical structures. In this study, heart models were selected owing to complex 3D relationships between components within the thoracic cavity. These heart models were personalized and customized to match the anatomical structure of each patient with congenital heart disease (CHD). The process of creating a 3D reconstruction from a patient’s computed tomography scan is shown in Multimedia Appendix 1. The 3D segmented models were constructed for an interrupted aortic arch (Multimedia Appendix 2), Ebstein anomaly (Multimedia Appendix 3), transposition of the great arteries (Multimedia Appendix 4), and major aortopulmonary collateral arteries (Multimedia Appendix 5). The segmented and processed images were then integrated into various tools: VR via Oculus Quest 2 (Meta), AR via HoloLens 2 (Microsoft Corp), and 3DP for physical modeling. In the final week, students presented on the future of medical education and clinical environments, drawing upon tools from the first 3 weeks of the course. In the curriculum management process, faculty members specializing in anatomy education oversaw the development of these tools and modalities. In addition, the course in which these tools were introduced was typically taught by not only anatomy experts, who use these technologies effectively in anatomy education, but also software developers. Content developers in the course worked with students to create CHD content.

**Table 1:** Table of contents and schedule for the elective course at Seoul National University College of Medicine, 2023.

Week and time			Topic		Detailed content		Teaching method
**Week 1: Medical image–based AI^a^ technology (n=5)**							
	1:00-1:10 PM	Presurvey for students		N/A^b^		Survey	
	1:10-2:00 PM	Understanding medical image–based AI technology		N/A		Lecture	
	2:00-2:10 PM	Rest		N/A		N/A	
	2:10-2:30 PM	Use of AI technology in medical imaging; 3DP^c^		N/A		Lecture	
	2:30-2:40 PM	Rest		N/A		N/A	
	2:40-3:00 PM	Use of AI technology in medical imaging; VR^d^, AR^e^, and MR^f^		N/A		Lecture	
	3:00-3:10 PM	Rest		N/A		N/A	
	3:10-4:50 PM	Learning the functions of medical image processing software		N/A		Lecture and hands-on practice	
	4:50-5:00 PM	Course wrap-up		N/A		N/A	
**Week 2: AI segmentation using medical image processing software (n=15; 4 groups)**							
	1:00-1:50 PM	AI segmentation using medical image processing software		AI segmentation on cases of CHD^g^ patients, including IAA^h^, Ebstein anomaly, TGA^i^, and MAPCA^j^		Hands-on practice	
	1:50-2:00 PM	Rest		N/A		N/A	
	2:00-2:50 PM	AI segmentation using medical image processing software		AI segmentation on the abovementioned cases		Hands-on practice	
	2:50-3:00 PM	Rest		N/A		N/A	
	3:00-3:50 PM	AI segmentation using medical image processing software		AI segmentation on the abovementioned cases		Hands-on practice	
	3:50-4:00 PM	Rest		N/A		N/A	
	4:00-4:50 PM	AI segmentation using medical image processing software		AI segmentation on the abovementioned cases		Hands-on practice	
	4:50-5:00 PM	Course wrap-up		N/A		N/A	
**Week 3: VR, AR, and 3DP experience (n=15; 4 groups)**							
	1:00-4:30 PM	VR experience		Anatomy structures Pediatric CHD model, including IAA, Ebstein anomaly, TGA, and MAPCA (patient-specific model) Digestive process Respiratory process Muscle movement		Group rotation experience	
	1:00-4:30 PM	AR experience		Pediatric CHD model, including IAA, Ebstein anomaly, TGA, and MAPCA (patient-specific model) Kidney cancer model Brain tumor model		Group rotation experience	
	1:00-4:30 PM	3DP experience		Pediatric CHD model, including IAA, Ebstein anomaly, TGA, and MAPCA (patient-specific model) Simulator model for surgery training		Group rotation experience	
	1:00-4:30 PM	3DP lab tour		3DP lab Production process lab		Group rotation experience	
	4:30-4:50 PM	Rest		N/A		N/A	
	4:50-5:00 PM	Course wrap-up		N/A		N/A	
**Week 4: Presentation (n=15; 4 groups)**							
	1:00-1:30 PM	Group 1: Presentation and discussion		N/A		Presentation and discussion	
	1:30-1:40 PM	Rest		N/A		N/A	
	1:40-2:10 PM	Group 2: Presentation and discussion		N/A		Presentation and discussion	
	2:10-2:20 PM	Rest		N/A		N/A	
	2:20-2:50 PM	Group 3: Presentation and discussion		N/A		Presentation and discussion	
	2:50-3:00 PM	Rest		N/A		N/A	
	3:00-3:30 PM	Group 4: Presentation and discussion		N/A		Presentation and discussion	
	3:30-4:00 PM	Rest		N/A		N/A	
	4:00-4:20 PM	Postsurvey for students and developers		N/A		Survey	
	4:20-4:40 PM	Group photo		N/A		N/A	
	4:40-5:00 PM	Course wrap-up		N/A		N/A	

^a^AI: artificial intelligence.

^b^N/A: not applicable.

^c^3DP: 3D printing.

^d^VR: virtual reality.

^e^AR: augmented reality.

^f^MR: mixed reality.

^g^CHD: congenital heart disease.

^h^IAA: interrupted aortic arch.

^i^TGA: transposition of the great arteries.

^j^MAPCA: major aortopulmonary collateral arteries.

### Devices and Software

This study employed a virtual dissection table (MDBOX, MEDICAL IP Co, Ltd), a VR headset (Oculus Quest 2), an AR headset (HoloLens 2), and medical image processing software (MEDIP PRO, MEDICAL IP Co, Ltd).

### Student Pre- and Postsurveys

The students’ survey consisted of pre- and postsurveys. The presurvey included questions about demographic information and prior experience with 3D medical technologies. The majority of previous studies used 5-point Likert scale–based questionnaires to assess user satisfaction, device usability, perceived engagement, and the influence on anatomy education [49]. Therefore, in the postsurvey, students used a 5-point Likert-type scale to indicate their overall satisfaction and satisfaction levels with VR, AR, and 3DP across 7 categories, which were organized into 2 subscales each (Multimedia Appendix 6). The 7 categories included esthetics, understanding of the concept, reality, spatial ability, immersion, continuous use intention, and future use. The use of new technologies in education is considered to be about experiences that include esthetic enjoyment as well [50]. Moreover, well-designed and esthetically pleasing content is more likely to engage users and improve their overall experience [51]. Clarity and comprehensibility of the content are crucial for users to effectively grasp concepts. 3D models provide users with the flexibility to explore structures from desired angles, thereby facilitating deeper comprehension and learning [52]. In education, aspects of spatial ability can be enhanced through experience, suggesting the potential for facilitating users’ learning of 3D structures [53]. Previous studies have found a correlation between spatial abilities and assessment of anatomy knowledge, with spatial training being shown to improve spatial abilities [54]. In these technology-rich educational environments, immersion has been identified as one of the primary drivers of student learning [55]. Continuous use intention is an important factor for the successful adoption of technology [56]. Additionally, satisfaction with technologies expected to be used in clinical areas or their potential for substitution was also investigated. To further explore students’ thoughts and insights through their presentations, students were asked questions about their expectations regarding the use of these technologies in various situational scenarios in the future. These questionnaires were prepared using Google Forms (Google LLC).

### Developer Survey

The developers took a single survey, which included demographic information, department affiliation, and years of professional experience. Similar to the student postsurvey, developers rated their overall satisfaction and satisfaction levels with VR, AR, and 3DP using a 5-point Likert-type scale across the same 7 categories organized into 2 subscales each. The survey also inquired about their expectations regarding the integration of these technologies into various medical settings within 5 years. Furthermore, the survey included questions exploring only developers’ perspectives on the advantages and challenges of VR, AR, MR, and 3DP. These questionnaires were prepared using Google Forms (Google LLC).

### Statistical Analysis

Statistical analyses were performed using SPSS software, version 26 (IBM Corp) and Prism, version 9 (GraphPad). Differences in satisfaction levels between students and developers for VR, AR, and 3DP were assessed using independent *t* tests. Statistical significance was determined at *P*<.05. Owing to the possibility of a type Ⅰ (false positive) error resulting from the multiple comparison analyses, we applied Bonferroni correction. After Bonferroni correction, most of the associations were not considered significant, with the adjusted significance level set at *P*<.003.

### Ethical Considerations

This study was approved by the Institutional Review Board of Seoul National University College of Medicine (E-2307-030-1447). The study was entirely retrospective (using existing student and developer surveys), and the requirement for informed consent was waived.

## Results

### Participants

In this survey study, we recruited 15 first-year medical students and 26 software and content developers who participated in a 4-week elective course that combined lectures and hands-on sessions. The data collection started on May 22, 2023, and ended on June 19, 2023.

### Demographic and Other Characteristics of Medical Students

The demographic and other characteristics of the 15 medical students are provided in Table 2. Their mean age was 21.5 (SD 1.5) years, and there were 12 (80%) male students and 3 (20%) female students. All students were familiar with VR, and 14 (93%) students were aware of AR and MR. However, 14 (93%) students had difficulty distinguishing between these technologies. Regarding VR content, 11 (73%) students engaged with it 1-3 times a month, with 8 (73%) engaging for educational purposes and 5 (46%) engaging for gaming. AR content was less frequent, with 5 (33%) students experiencing it 1-2 times a year, mainly in gaming (4/5, 80%) and education (2/5, 40%). 3DP content was used 1-2 times a year for educational purposes by 9 (60%) students. Moreover, 11 (73%) students used VR-based medical content, with 6 (55%) students focusing on heart-related content (heart VR education was previously provided in the anatomy curriculum) and 5 (46%) using unknown content. None had prior experience with AR-based medical content, but 2 (13%) students had experience with 3DP-based medical content. Of these 2 students, 1 (50%) used a pediatric cardiac model and 1 (50%) used content of unknown nature.

**Table 2:** Demographic and other characteristics of medical students (n=15).

Characteristic		Value
Age (years), mean (SD)		21.5 (1.5)
**Gender, n (%)**		
	Male	12 (80)
	Female	3 (20)
**Please select all the options you have heard of among VR^a^, AR^b^, MR^c^, and 3DP^d^, n (%)**		
	VR	15 (100)
	AR	14 (93)
	MR	14 (93)
**Can you distinguish between VR, AR, and MR? n (%)**		
	No	14 (93)
	Yes	1 (7)
**Have you ever experienced VR content? n (%)**		
	No	4 (27)
	Yes	11 (73)
**If you have experienced VR content, how often did you experience it?^e^, n (%)**		
	Everyday	0 (0)
	3-4 times a week	0 (0)
	1-2 times a week	0 (0)
	1-3 times a month	11 (100)
	1-2 times a year	0 (0)
**If you have experienced VR content, please select all the experiences you had^e^, n (%)**		
	Game	5 (46)
	Travel	0 (0)
	Movies or television shows	0 (0)
	Music (eg, concerts and music videos)	0 (0)
	Education	8 (73)
	Art galleries	0 (0)
**Have you ever experienced AR content? n (%)**		
	No	10 (67)
	Yes	5 (33)
**If you have experienced AR content, how often did you experience it?^e^, n (%)**		
	Everyday	0 (0)
	3-4 times a week	0 (0)
	1-2 times a week	0 (0)
	1-3 times a month	0 (0)
	1-2 times a year	5 (100)
**If you have experienced AR content, please select all the experiences you had^e^, n (%)**		
	Game	4 (80)
	Travel	0 (0)
	Movies or television shows	0 (0)
	Music (eg, concerts and music videos)	0 (0)
	Education	2 (40)
	Art galleries	0 (0)
**Have you ever experienced 3DP content? n (%)**		
	No	6 (40)
	Yes	9 (60)
**If you have experienced 3DP content, how often did you experience it?^e^, n (%)**		
	Everyday	0 (0)
	3-4 times a week	0 (0)
	1-2 times a week	0 (0)
	1-3 times a month	0 (0)
	1-2 times a year	9 (100)
**If you have experienced 3DP content, please select all the experiences you had^e^, n (%)**		
	Game	0 (0)
	Travel	0 (0)
	Movies or television shows	0 (0)
	Music (eg, concerts and music videos)	0 (0)
	Education	9 (100)
	Art galleries	0 (0)
**Have you ever experienced VR-based medical content?** **n (%)**		
	No	4 (27)
	Yes	11 (73)
**If you have experienced VR-based medical content, what is the name of the content? (If unknown, please write “unknown”)^e^, n (%)**		
	Heart	6 (55)
	Unknown	5 (46)
**Have you ever experienced AR-based medical content? n (%)**		
	No	15 (100)
	Yes	0 (0)
**Have you ever experienced 3DP-based medical content?** **n (%)**		
	No	13 (87)
	Yes	2 (13)
**If you have experienced 3DP-based medical content, what is the name of the content? (If unknown, please write “unknown”)^e^, n (%)**		
	Pediatric cardiac model	1 (50)
	Unknown	1 (50)

^a^VR: virtual reality.

^b^AR: augmented reality.

^c^MR: mixed reality.

^d^3DP: 3D printing.

^e^Only the subgroup of students who experienced either VR, AR, or 3DP.

### Demographic and Other Characteristics of Developers

The demographic and other characteristics of the 26 developers are shown in Table 3. Their mean age was 28.2 (SD 4.5) years, and there were 7 (27%) male developers and 19 (73%) female developers. There was no bias in their expertise. The developers were individuals with backgrounds in software and content development and had various degrees and majors (Multimedia Appendix 7). Among the 26 developers, 15 (58%) were associated with VR, 4 (15%) with AR, and 10 (39%) with 3DP. Regarding their years of professional experience, most developers had 1 year of experience (9/26, 35%), followed by less than 1 year of experience (4/26, 15%); 4 and 5 years of experience (each 3/26, 12%); 2, 3, and 8 years of experience (each 2/26, 8%); and 7 years of experience (1/26, 4%).

**Table 3:** Demographic and other characteristics of developers (n=26).

Characteristic			Value
Age (years), mean (SD)			28.2 (4.5)
**Gender, n (%)**			
	Male	7 (27)	
	Female	19 (73)	
**Which departments are you affiliated with? n (%)**			
	VR^a^	15 (58)	
	AR^b^	4 (15)	
	3DP^c^	10 (39)	
**How many years of experience do you have? n (%)**			
	No experience	0 (0)	
	Less than 1 year	4 (15)	
	1 year	9 (35)	
	2 years	2 (8)	
	3 years	2 (8)	
	4 years	3 (12)	
	5 years	3 (12)	
	6 years	0 (0)	
	7 years	1 (4)	
	8 years	2 (8)	
	9 years	0 (0)	
	More than 10 years	0 (0)	

^a^VR: virtual reality.

^b^AR: augmented reality.

^c^3DP: 3D printing.

### Levels of Satisfaction With VR, AR, and 3DP Between Medical Students and Developers

**Figure 4:**
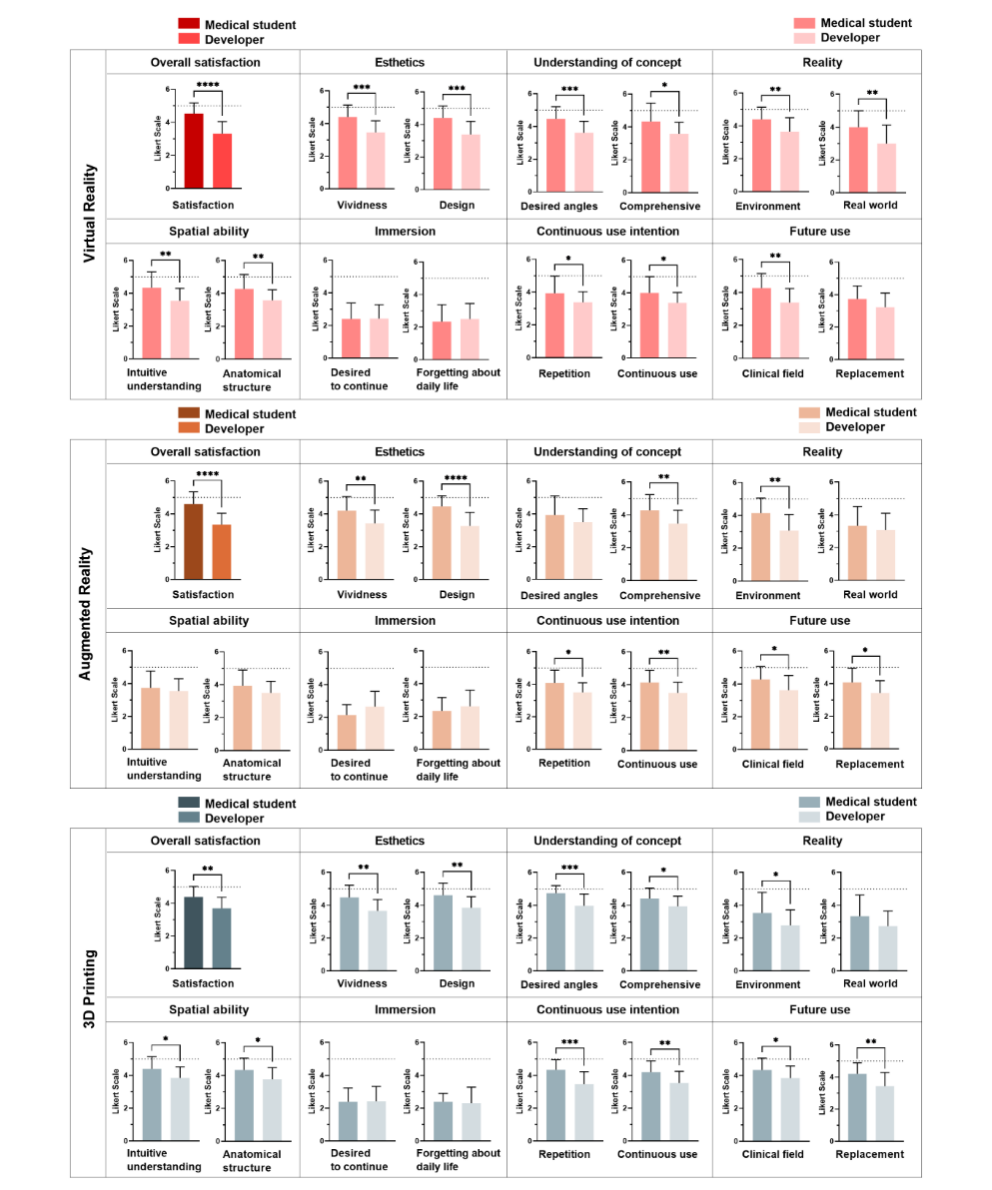
Medical students' (n=15) and developers' (n=26) levels of satisfaction with virtual reality, augmented reality, and 3D printing have been ranked on a 5-point Likert-type scale (1=very dissatisfied to 5=very satisfied). Participants were asked about their overall satisfaction with virtual reality, augmented reality, and 3D printing, as well as their satisfaction with 7 categories organized into 2 subscales. *P<.05, **P<.01, ***P<.001, ****P<.0001.

Levels of satisfaction with VR, AR, and 3DP among medical students and developers are shown in Figure 4 and Tables S1-S3 in Multimedia Appendix 8. The satisfaction results are presented in terms of overall satisfaction and the following 7 categories: esthetics, understanding of the concept, reality, spatial ability, immersion, continuous intention, and future use. Specifically, 2 questions were included in each category. In terms of esthetics, the vividness and design of the content were considered. For understanding of the concept, questions assessed how easily participants understood the content and if they were able to learn effectively. Reality focused on whether participants felt a sense of realism within the content and its surroundings. Spatial ability questions evaluated participants’ satisfaction to intuitively grasp the structures and understand the relationships between different structures. Continuous use intention investigated participants’ desire to repeatedly engage with the content and continue its use. Future use included specific inquiries about the potential application of these technologies in clinical settings and their potential to replace conventional methods.

Overall satisfaction with VR, AR, and 3DP was significantly higher among students than among developers. There was no significant difference in satisfaction with immersion in VR, AR, and 3DP between medical students and developers. In the case of VR (Figure 4 and Table S1 in Multimedia Appendix 8), the satisfaction levels of students were significantly higher than those of developers in esthetics (vividness: 4.52 vs 3.31; design: 4.40 vs 3.38), understanding of the concept (desired angles: 4.47 vs 3.62; comprehensive: 4.33 vs 3.58), reality (environment: 4.40 vs 3.65; real world: 4.00 vs 3.00), spatial ability (intuitive understanding: 4.33 vs 3.54; spatial perception: 4.27 vs 3.58), continuous use intention (repetition: 3.93 vs 3.39; continuous use: 4.00 vs 3.39), and future use (clinical field: 4.27 vs 3.39). However, after Bonferroni correction to adjust for multiple variables, only overall satisfaction (*P*<.001), esthetics (vividness and design, *P*<.001), and understanding of the concept (desired angles, *P*<.001) remained statistically significant.

Similarly, in the case of AR (Figure 4 and Table S2 in Multimedia Appendix 8), the satisfaction levels of students were significantly higher than those of developers in esthetics (vividness: 4.20 vs 3.42; design: 4.47 vs 3.27), understanding of the concept (comprehensive: 4.27 vs 3.46), reality (environment: 4.13 vs 3.08; real world: 4.00 vs 3.00), continuous use intention (repetition: 4.07 vs 3.50; continuous use: 4.13 vs 3.50), and future use (clinical field: 4.27 vs 3.61; replacement: 4.07 vs 3.42). However, after Bonferroni correction to adjust for multiple variables, only overall satisfaction (*P*<.001), esthetics (design, *P*<.001), and reality (environment, *P*=.002) remained statistically significant.

In the case of 3DP (Figure 4 and Table S3 in Multimedia Appendix 8), the satisfaction levels of students were significantly higher than those of developers in esthetics (vividness: 4.47 vs 3.65; design: 4.60 vs 3.85), understanding of the concept (desired angles: 4.73 vs 3.96; comprehensive: 4.40 vs 3.92), reality (environment: 3.53 vs 2.77), spatial ability (intuitive understanding: 4.40 vs 3.85; spatial perception: 4.33 vs 3.77), continuous use intention (repetition: 4.33 vs 3.46; continuous use: 4.20 vs 3.54), and future use (clinical field: 4.33 vs 3.85; replacement: 4.20 vs 3.42). However, after Bonferroni correction to adjust for multiple variables, only overall satisfaction (*P*<.001), esthetics (vividness, *P*=.001; design, *P*=.002), understanding of the concept (desired angles, *P*<.001), and continuous use intention (repetition, *P*<.001) remained statistically significant.

### Anticipations for the Future Use of VR, AR, MR, and 3DP Between Medical Students and Developers

Future usage expectations of VR, AR, MR, and 3DP between medical students and developers are illustrated in Multimedia Appendix 9. Students expected VR and AR to be the most frequently used technologies in medical education (8/15, 53% and 5/15, 33%, respectively), while MR and 3DP were less anticipated. In contrast, 10 (38%) developers predicted MR to be the most widely used technology, followed by 3DP, with VR and AR having low expected use. In educating residents, 7 (47%) students anticipated 3DP to be the most widely used technology, followed by MR, VR, and AR. Moreover, 11 (42%) developers expected MR to be the dominant technology, followed by 3DP, AR, and VR.

For individual surgical planning, 7 (47%) students expected 3DP to be the most widely used technology, followed by VR, AR, and MR. Moreover, 11 (42%) developers anticipated MR to be the most widely used technology, followed by 3DP, VR, and AR. In collaborative surgical planning, 6 (46%) students anticipated AR to be the most widely used technology, followed by MR, VR, and 3DP. Moreover, 12 (46%) developers expected MR to be the dominant technology, followed by 3DP, AR, and VR.

For surgical procedures on patients, 8 (53%) students expected AR to be the most widely used technology, followed by VR, MR, and 3DP. Moreover, 14 (52%) developers anticipated MR to be the most widely used technology. In explaining medical information to patients’ caregivers, 13 (87%) students anticipated 3DP to be the most widely used technology, followed by VR and MR. Moreover, 10 (38%) developers anticipated 3DP to be the most widely used technology, followed by MR, AR, and VR.

### Developers’ Perceptions of the Advantages and Current Challenges in VR, AR, MR, and 3DP

The developers’ views on the advantages and current challenges of VR, AR, MR, and 3DP are presented in Multimedia Appendix 10. Regarding VR, developers highlighted immersion (11/26, 42%) and the expansiveness of space (8/26, 31%) as key advantages. However, none reported providing experiences similar to real environments as a VR advantage. The main challenge was hardware performance (4/26, 31%), followed by a lack of proactive content production, user-friendliness, and pricing and health issues.

Regarding AR, developers considered engagement in AR (11/26, 42%) as its main advantage, followed by recognizing interactivity and appreciating its ability to provide experiences similar to real environments. None of them reported refining information in AR as an advantage. The main challenge was hardware performance (10/26, 38%), followed by user-friendliness, a lack of proactive content production, and issues related to price and health.

Regarding MR, developers highlighted the integration of real and virtual spaces as the primary advantage (13/26, 50%), with 19% (5/26) noting the provision of realistic virtual spaces. Interactivity and the innovativeness of the experience were also reported. None reported refining information as an advantage. Hardware performance was the primary concern (11/26, 42%), followed by user-friendliness, a lack of proactive content production, and issues related to price and health.

Lastly, regarding 3DP, the primary advantage was the possibility of customized product manufacturing (21/26, 81%). Some developers recognized high accuracy and texture fidelity, and reported increased creative freedom and fast product production. However, none mentioned new design possibilities as an advantage. The primary challenge was manufacturing costs (18/26, 69%), followed by long printing times, difficulty in creating complex models, limitations of materials, and low durability of printed objects (all 2/26, 8%).

## Discussion

### Overview

Our study aimed to investigate the gaps in perspectives between medical students and developers regarding satisfaction and the most anticipated future use of 3D technologies in medical education. This study offers insights into the differences in satisfaction levels between medical students and developers. This study also provides insights into how anticipations for the use of these technologies differ between medical students and developers across different situational scenarios, as well as how these technologies might be used in specific specialties or areas of medicine. Additionally, this study sheds light on developers’ viewpoints regarding the advantages and challenges associated with these technologies, with the aim of understanding their applicability and limitations in industrial settings.

### Principal Findings

The results of this study provide unique evidence that medical students have a higher level of overall satisfaction than developers across VR, AR, and 3DP technologies (Figure 4), which implies that there might be a stronger alignment between the satisfaction and experiences of students with these technologies. It is also noteworthy that medical students were more satisfied than developers with esthetics and the intention to continue use among the 7 categories in VR, AR, and 3DP technologies (Figure 4). We assume that students had positive experiences with the liveliness of technology and design through this course, and based on this experience, they will have high expectations for the future in terms of the vividness and design of these technologies. We believe that students had higher satisfaction with the intention to continue use compared to developers because, as users, they perceive that these technologies yield greater learning effectiveness when used repetitively in the learning environment.

The extent of satisfaction difference between medical students and developers varied across VR, AR, and 3DP. Regarding VR, the difference in satisfaction between medical students and developers was the greatest for esthetics and conceptual understanding (desired angle). This difference could be attributed to the experience students had during the course. Students would have experienced higher satisfaction by interacting with the CHD model they created in the course, such as by rotating the CHD model they created to the desired angle in virtual space, understanding the structure, and applying color. We speculate that esthetics showed the largest extent of difference in AR because students experienced higher satisfaction than developers as the design made it easy for them to recognize and interact with the CHD models they created when presented in a real-world environment. Regarding 3DP, the difference in satisfaction was the greatest for concept understanding (desired angle) and continued use intention (repetition). The patient-customized CHD model could be rotated at any desired angle in real space, and it is assumed that continuous repetition helps students understand complex anatomy.

This study found that when comparing all categories with each technology, medical students were more satisfied than developers in reality (real world) only within VR (Figure 4). Based on the findings of the study, it can be inferred that VR technology, particularly in its current state of hardware development, offers medical students a more realistic and satisfying experience compared to developers. On the other hand, developers may be less satisfied with the current state of technology when considering both the current state of technology and the potential for future technological advances in the industry. This may be because developers who actively participate in the industry are aware of the substantial difficulty needed to increase the realism of VR. In addition, AR showed no significant difference in satisfaction between medical students and developers in terms of understanding of the concept (desired angles) and spatial ability (Figure 4). Therefore, we conclude that VR might offer a more immersive and satisfying experience for medical students based on current hardware technologies, while AR appears to offer a more balanced perception in terms of conceptual understanding and spatial awareness.

One of the key findings of our study was that there was a gap in anticipations between medical students and developers in 6 situational scenarios regarding the anticipated future use of technology, with the exception of 1 situational scenario (Multimedia Appendix 9). Situational scenarios in which the perspectives differed included educating medical students and residents, individual and collaborative surgical planning, and performing surgery on a patient. In these scenarios, developers perceived MR as a more promising technology. In contrast, medical students perceived VR primarily for student education, 3DP for resident education and individual surgical planning, and AR for collaborative surgical planning and performing surgery on a patient. This discrepancy between the 2 groups is likely from variations in exposure and practical experience with these technologies. Although students experienced VR, AR, and 3DP in their elective course, they were not exposed to MR. Despite the limited exposure to MR among medical students, we can speculate on their perspectives regarding its future use based on their experiences. Medical students may see VR primarily for undergraduate education because of its immersive and interactive nature, allowing for realistic simulations [4]. They may see 3DP as beneficial for resident training and individual surgical planning because of its hands-on nature, allowing them to create physical models that can enhance their understanding of anatomical structures and medical conditions. This perception could be attributed to its potential for customized product manufacturing, which could potentially facilitate clearer communication of medical information to nonexperts. This result is consistent with the results of previous studies, which tended to report positive correlations between the use of 3DP and resident education and explanation to patient caregivers [28,57-59]. Regarding AR, medical students may see it as suitable for collaborative surgical planning and performing surgery on a patient because of its potential to overlay digital information onto the real surgical environment, providing surgeons with real-time guidance and information during procedures. This aligns with existing research on its benefits in specific surgical procedures, such as spine and orthopedic surgeries [60,61]. Our study implied a potential interest in exploring the application of AR in the surgical field among medical students. These findings of our study emphasize the need to align technological advancements with the expectations of both medical students and developers. By meeting the expectations of both groups, these technologies can be smoothly integrated into medical education.

This study highlights an interesting alignment in perspectives between medical students and developers, particularly in the situational scenario of explaining to a patient’s caregiver. In this scenario, both groups showed potential interest in 3DP for conveying complex medical information to a patient’s caregiver. Additionally, considering that students and developers in this study participated in creating patient-specific 3D-printed heart models and that students gave group presentations with this technology, it can be inferred that personalized 3D-printed models are helpful in patients’ caregiver communication. Previous studies demonstrated that the use of personalized 3DP models can further enhance patient understanding by providing tailored visual representations of individual patient anatomy and medical conditions [57,62,63].

This exploration of varying expectations will offer insights into how these technologies are anticipated to shape the future of medical training, patient care, and medical research. Several potential applications can be envisioned based on our research findings. In medical education for medical students and patients, VR, MR, and 3DP could be used for anatomy learning, medical research, simulation training, and procedural skills practice. VR can be used for virtual simulations of procedures like suturing, catheterization, and intubation, as well as clinical scenarios like patient assessments and diagnostic procedures. MR is expected to enhance anatomical learning and hands-on procedural training by combining virtual and real-world elements. It will overlay digital models onto physical specimens, which are generated from 3DP, and enable realistic simulations with them. Our speculation involves the use of AR, MR, and 3DP in surgical planning. Based on patient-specific medical imaging data, it is anticipated that surgeons will use patient-specific 3DP models to physically review and plan surgical approaches before the actual surgery. Alternatively, they may use AR and VR for surgical simulations to plan the procedure in advance. We also speculate that AR and MR will be used in specialties, such as neurosurgery, cardiovascular surgery, etc. Surgeons can use AR holograms of the heart or lungs to visualize complex cardiac anatomy during surgery and to orient and localize the target tumors or lesions. MR-guided interventions can facilitate minimally invasive procedures, such as transcatheter valve replacement, by providing real-time imaging guidance and navigation. VR and 3DP are expected to benefit patient care. We speculate that VR will help manage pain and reduce stress during treatment, while 3DP will allow for personalized models, improving the understanding of patient conditions and treatments.

While the majority of developers perceived the inherent capabilities of VR, AR, and MR technologies as strengths, an interesting aspect of our findings is that none of them mentioned providing experiences similar to real environments as a strength of VR technology or cited the refinement of information in AR technology (Multimedia Appendix 10). We infer that developers perceive providing experiences similar to real environments and refinement of information as technically challenging at present or as areas requiring further development and thus fail to recognize the benefits of each technology. In fact, modeling of environments, especially in the medical field, requires the creation of high-quality 3D objects [64]. Reaching highly realistic and natural photorealistic rendering and animations in full 3D can be exceedingly challenging and costly in terms of both time and money [65]. Therefore, we speculate that in addition to the advantages of each technology that developers currently recognize, additional improvement and development are needed for aspects of each technology that developers are not aware of at present.

This study identified a concern regarding developers’ limited attention to health issues, although there is a high prevalence of computer vision syndrome as an occupational disease in the 21st century (Multimedia Appendix 10). Additionally, the focus of developers on hardware performance over health issues indicates concerning results where technical priorities overshadow user well-being. To address this, developers must adopt a more holistic approach that balances technical advancements with user safety. This includes integrating health considerations into the design and development process, implementing safety features, and conducting thorough user testing to mitigate health-related issues [66]. By prioritizing both technical excellence and user welfare, developers will enhance the overall ethical standards of these technologies and contribute to a safer and more responsible technological landscape.

Regarding 3DP, our results showed that developers perceived the practical aspect of manufacturing customized products as an advantage of 3DP over the creative aspect of new design possibilities. This finding is consistent with previous studies reporting that customization allows for printing parts with geometries tailored to each print, which can be particularly useful in patient-specific fabrications for personalized medicine, where the layout matches a specific patient’s anatomy [67]. As a significant challenge, the developers in this study and several other studies recognized high manufacturing costs [68]. However, in general, 3DP has been applied in the medical field. Therefore, this study suggests the need for continued research and development efforts aimed at optimizing the cost-effectiveness of 3DP technology without compromising on its advantages.

### Limitations

This study has several limitations. First, this study was conducted in a single institution with a relatively small sample size. Further studies should be conducted and compared across multiple medical schools. Second, this study was limited by the exclusive focus on first-year medical students and developers involved in the course. While this provided valuable insights into the perspectives of these specific groups, the exclusion of residents, fellows, and senior medical professionals may limit the generalizability and applicability of our findings. Participants from diverse backgrounds should be included in further studies. Third, owing to the voluntary nature of student participation in the course, participant selection was not conducted. Consequently, our study results may be influenced by the higher proportion of male individuals than female individuals in the student group, potentially resulting in a dominance of male perspectives in the outcomes. Fourth, students having difficulty distinguishing between VR, AR, and MR experiences may have influenced the accuracy of the self-reported engagement with these technologies. Future studies should consider incorporating educational interventions to enhance students’ understanding of various immersive technologies before administering surveys on technology usage. Lastly, it was not possible to validate the instruments used in this study, and we used a limited number of questionnaire items to measure the levels of students’ and developers’ satisfaction with the elective course. Despite these limitations, this study might help to understand differences in satisfaction levels between medical students and developers, as well as discrepancies in their perceptions of future technological advancements.

### Conclusion

The roadblock for better integration of VR, AR, MR, and 3DP technologies in medical education is the gap in satisfaction levels and future anticipations between medical students and developers. Our study found that VR, AR, and 3DP technologies showed differences in satisfaction levels in the categories of esthetics and continuous use intention. In particular, in VR, differences in satisfaction levels regarding reality (real world) emerged as a major obstacle to integration into medical education. Medical students and developers had different anticipations of the future use of technology regarding education, surgical planning, and surgery. Furthermore, insights from industry developers indicated that hardware performance poses a challenge for VR, AR, and MR, while high manufacturing cost is the primary concern for 3DP. Recognizing and understanding these discrepancies and current challenges can help developers tailor their strategies and innovations to better meet the expectations of technology users.

## Appendix

Multimedia Appendix 1 The process of creating a 3D reconstruction from a patient's computed tomography scan.

Multimedia Appendix 2 The heart of a patient with an interrupted aortic arch.

Multimedia Appendix 3 The heart of a patient with Ebstein anomaly.

Multimedia Appendix 4 The heart of a patient with transposition of the great arteries.

Multimedia Appendix 5 The heart of a patient with major aortopulmonary collateral arteries.

Multimedia Appendix 6 A survey on satisfaction with virtual reality, augmented reality, and 3D printing technologies.

Multimedia Appendix 7 The educational background of the software and content developers in this study.

Multimedia Appendix 8 Average satisfaction differences in virtual reality, augmented reality, and 3D printing between students and developers.

Multimedia Appendix 9 Medical students' (n=15) and developers' (n=26) anticipation of the use of technologies in various medical contexts within 5 years. The pie charts present percentages.

Multimedia Appendix 10 Developers' perceptions of the advantages and current challenges of virtual reality, augmented reality, mixed reality, and 3D printing.

## Abbreviations

3DP: 3D printing
AI: artificial intelligence
AR: augmented reality
CHD: congenital heart disease
MR: mixed reality
VR: virtual reality

## Data Availability

The data used or analyzed in this study are available from the corresponding author upon reasonable request.

## References

[1] Aliwi I, Schot V, Carrabba M, Duong P, Shievano S, Caputo M, Wray J, de Vecchi A, Biglino G (2023). The role of immersive virtual reality and augmented reality in medical communication: a scoping review. J Patient Exp.

[2] Tasneem I, Ariz A, Bharti D, Haleem A, Javaid M, Bahl S (2021). 3D printing technology and its significant applications in the context of healthcare education. J Ind Intg Mgmt.

[3] Gerup J, Soerensen CB, Dieckmann P (2020). Augmented reality and mixed reality for healthcare education beyond surgery: an integrative review. Int J Med Educ.

[4] Radianti J, Majchrzak T, Fromm J, Wohlgenannt I (2020). A systematic review of immersive virtual reality applications for higher education: Design elements, lessons learned, and research agenda. Computers & Education.

[5] Valls-Esteve A, Adell-Gómez N, Pasten A, Barber I, Munuera J, Krauel L (2023). Exploring the potential of three-dimensional imaging, printing, and modeling in pediatric surgical oncology: a new era of precision surgery. Children (Basel).

[6] Venugopal JP, Subramanian AAV, Peatchimuthu J (2023). The realm of metaverse: a survey. Computer Animation & Virtual.

[7] Rokhsaritalemi S, Sadeghi-Niaraki A, Choi S (2020). A review on mixed reality: current trends, challenges and prospects. Applied Sciences.

[8] Venkatesan M, Mohan H, Ryan J, Schürch C, Nolan G, Frakes D, Coskun A (2021). Virtual and augmented reality for biomedical applications. Cell Rep Med.

[9] Sugimoto M, Takenoshita S, Yasuhara H (2021). Extended reality (XR:VR/AR/MR), 3D printing, holography, A.I., radiomics, and online VR tele-medicine for precision surgery. Surgery and Operating Room Innovation.

[10] Ghazi AE, Teplitz BA (2020). Role of 3D printing in surgical education for robotic urology procedures. Transl Androl Urol.

[11] Jiang H, Vimalesvaran S, Wang J, Lim K, Mogali S, Car L (2022). Virtual reality in medical students' education: scoping review. JMIR Med Educ.

[12] Karbasi Z, Niakan Kalhori S (2020). Application and evaluation of virtual technologies for anatomy education to medical students: a review. Med J Islam Repub Iran.

[13] Zhao J, Xu X, Jiang H, Ding Y (2020). The effectiveness of virtual reality-based technology on anatomy teaching: a meta-analysis of randomized controlled studies. BMC Med Educ.

[14] Tang Y, Chau K, Kwok A, Zhu T, Ma X (2022). A systematic review of immersive technology applications for medical practice and education - trends, application areas, recipients, teaching contents, evaluation methods, and performance. Educational Research Review.

[15] Durrani S, Onyedimma C, Jarrah R, Bhatti A, Nathani KR, Bhandarkar AR, Mualem W, Ghaith AK, Zamanian C, Michalopoulos GD, Alexander AY, Jean W, Bydon M (2022). The virtual vision of neurosurgery: how augmented reality and virtual reality are transforming the neurosurgical operating room. World Neurosurg.

[16] Dhar P, Rocks T, Samarasinghe R, Stephenson G, Smith C (2021). Augmented reality in medical education: students' experiences and learning outcomes. Med Educ Online.

[17] Cellina M, Cè M, Alì M, Irmici G, Ibba S, Caloro E, Fazzini D, Oliva G, Papa S (2023). Digital twins: the new frontier for personalized medicine?. Applied Sciences.

[18] Xie B, Liu H, Alghofaili R, Zhang Y, Jiang Y, Lobo F, Li C, Li W, Huang H, Akdere M, Mousas C, Yu L (2021). A review on virtual reality skill training applications. Front Virtual Real.

[19] Sánchez-Margallo J, Plaza de Miguel C, Fernández Anzules R, Sánchez-Margallo F (2021). Application of mixed reality in medical training and surgical planning focused on minimally invasive surgery. Front Virtual Real.

[20] Lungu AJ, Swinkels W, Claesen L, Tu P, Egger J, Chen X (2021). A review on the applications of virtual reality, augmented reality and mixed reality in surgical simulation: an extension to different kinds of surgery. Expert Rev Med Devices.

[21] Verhey J, Haglin J, Verhey E, Hartigan D (2020). Virtual, augmented, and mixed reality applications in orthopedic surgery. Int J Med Robot.

[22] Pregowska A, Osial M, Dolega-Dolegowski D, Kolecki R, Proniewska K (2022). Information and communication technologies combined with mixed reality as supporting tools in medical education. Electronics.

[23] Olatunji G, Osaghae O, Aderinto N (2023). Exploring the transformative role of 3D printing in advancing medical education in Africa: a review. Ann Med Surg (Lond).

[24] Karakurt I, Lin L (2020). 3D printing technologies: techniques, materials, and post-processing. Current Opinion in Chemical Engineering.

[25] Dod G, Jibhakate R, Walke P (2023). A review on 3D printing maxillofacial surgery: present work and future prospects. Materials Today: Proceedings.

[26] Ullah M, Hamayun S, Wahab A, Khan SU, Rehman MU, Haq ZU, Rehman KU, Ullah A, Mehreen A, Awan UA, Qayum M, Naeem M (2023). Smart technologies used as smart tools in the management of cardiovascular disease and their future perspective. Curr Probl Cardiol.

[27] Yan Q, Dong H, Su J, Han J, Song B, Wei Q, Shi Y (2018). A review of 3D printing technology for medical applications. Engineering.

[28] Cornejo J, Cornejo-Aguilar J, Vargas M, Helguero C, Milanezi de Andrade R, Torres-Montoya S, Asensio-Salazar J, Rivero Calle A, Martínez Santos J, Damon A, Quiñones-Hinojosa A, Quintero-Consuegra M, Umaña J, Gallo-Bernal S, Briceño M, Tripodi P, Sebastian R, Perales-Villarroel P, De la Cruz-Ku G, Mckenzie T, Arruarana V, Ji J, Zuluaga L, Haehn D, Paoli A, Villa J, Martinez R, Gonzalez C, Grossmann R, Escalona G, Cinelli I, Russomano T (2022). Anatomical engineering and 3D printing for surgery and medical devices: international review and future exponential innovations. Biomed Res Int.

[29] Patra A, Pushpa N, Ravi K, Abdel Meguid E, Mishall PL, Nation HL, Rea PM (2023). Visualization in anatomy education. Biomedical Visualisation. Advances in Experimental Medicine and Biology, vol 1406.

[30] Wickramasinghe N, Thompson BR, Xiao J (2022). The opportunities and challenges of digital anatomy for medical sciences: narrative review. JMIR Med Educ.

[31] Wixted CM, Peterson JR, Kadakia RJ, Adams SB (2021). Three-dimensional printing in orthopaedic surgery: current applications and future developments. JAAOS Glob Res Rev.

[32] Yun YH, Jo DH, Jeon SK, Kwon HY, Jeon YM, Shin DH, Choi HJ (2022). The impact of the modified schedules of anatomy education on students' performance and satisfaction: Responding to COVID-19 pandemic in South Korea. PLoS One.

[33] Pereda-Nuñez A, Manresa M, Webb SS, Pineda B, Espuña M, Ortega M, Rodríguez-Baeza A (2023). Pelvic + Anatomy: A new interactive pelvic anatomy model. Prospective randomized control trial with first-year midwife residents. Anat Sci Educ.

[34] Yan X, Zhu Y, Fang L, Ding P, Fang S, Zhou J, Wang J (2023). Enhancing medical education in respiratory diseases: efficacy of a 3D printing, problem-based, and case-based learning approach. BMC Med Educ.

[35] O'Connor M, Stowe J, Potocnik J, Giannotti N, Murphy S, Rainford L (2021). 3D virtual reality simulation in radiography education: The students' experience. Radiography (Lond).

[36] Dennler C, Bauer DE, Scheibler A, Spirig J, Götschi T, Fürnstahl P, Farshad M (2021). Augmented reality in the operating room: a clinical feasibility study. BMC Musculoskelet Disord.

[37] Paternò F, Wulf V (2017). New Perspectives in End-User Development.

[38] Shah H, Dingley S, Golder P (1994). Bridging the culture gap between users and developers. J Syst Manag.

[39] Subramanyam R, Weisstein FL, Krishnan MS (2010). User participation in software development projects. Commun. ACM.

[40] Melone N (1990). A theoretical assessment of the user-satisfaction construct in information systems research. Management Science.

[41] Ives B, Olson M, Baroudi J (1983). The measurement of user information satisfaction. Commun. ACM.

[42] Au N, Ngai EWT, Cheng TCE (2008). Extending the understanding of end user information systems satisfaction formation: An equitable needs fulfillment model approach. MIS Quarterly.

[43] Briggs R, Reinig B, Vreede G (2008). The yield shift theory of satisfaction and its application to the IS/IT domain. JAIS.

[44] Xiao W, Wang M, Mo J (2023). Factors influencing college teachers' adoption of live online teaching: a conditional process model of technology acceptance, user satisfaction and privacy concerns. Front Psychol.

[45] Elshami W, Taha M, Abuzaid M, Saravanan C, Al Kawas S, Abdalla M (2021). Satisfaction with online learning in the new normal: perspective of students and faculty at medical and health sciences colleges. Med Educ Online.

[46] Jedwab RM, Manias E, Redley B, Dobroff N, Hutchinson AM (2023). Impacts of technology implementation on nurses' work motivation, engagement, satisfaction and well-being: A realist review. J Clin Nurs.

[47] van Lente H, Verbeek PP, Slob A (2006). Expected behavior. User Behavior and Technology Development, vol 20.

[48] Arthur WB (1989). Competing technologies, increasing returns, and lock-in by historical events. The Economic Journal.

[49] Yi WS, Rouhi AD, Duffy CC, Ghanem YK, Williams NN, Dumon KR (2024). A systematic review of immersive virtual reality for nontechnical skills training in surgery. J Surg Educ.

[50] Cheng K, Tsai C (2012). Affordances of augmented reality in science learning: suggestions for future research. J Sci Educ Technol.

[51] Hemidli N Introduction to UI/UX design: key concepts and principles. Academia.

[52] Pujol S, Baldwin M, Nassiri J, Kikinis R, Shaffer K (2016). Using 3D modeling techniques to enhance teaching of difficult anatomical concepts. Acad Radiol.

[53] Sweeney K, Hayes JA, Chiavaroli N (2014). Does spatial ability help the learning of anatomy in a biomedical science course?. Anat Sci Educ.

[54] Langlois J, Bellemare C, Toulouse J, Wells GA (2020). Spatial abilities training in anatomy education: a systematic review. Anat Sci Educ.

[55] Georgiou Y, Kyza EA (2018). Relations between student motivation, immersion and learning outcomes in location-based augmented reality settings. Comput Hum Behav.

[56] Zhao H, Khan A (2021). The students' flow experience with the continuous intention of using online english platforms. Front Psychol.

[57] Ye Z, Dun A, Jiang H, Nie C, Zhao S, Wang T, Zhai J (2020). The role of 3D printed models in the teaching of human anatomy: a systematic review and meta-analysis. BMC Med Educ.

[58] Schlegel L, Ho M, Fields J, Backlund E, Pugliese R, Shine K (2022). Standardizing evaluation of patient-specific 3D printed models in surgical planning: development of a cross-disciplinary survey tool for physician and trainee feedback. BMC Med Educ.

[59] Youn JK, Park SJ, Choi Y, Han J, Ko D, Byun J, Yang H, Kim H (2023). Application of 3D printing technology for pre-operative evaluation, education and informed consent in pediatric retroperitoneal tumors. Sci Rep.

[60] Cofano F, Di Perna G, Bozzaro M, Longo A, Marengo N, Zenga F, Zullo N, Cavalieri M, Damiani L, Boges D, Agus M, Garbossa D, Calì C (2021). Augmented reality in medical practice: from spine surgery to remote assistance. Front Surg.

[61] Jud L, Fotouhi J, Andronic O, Aichmair A, Osgood G, Navab N, Farshad M (2020). Applicability of augmented reality in orthopedic surgery - a systematic review. BMC Musculoskelet Disord.

[62] Zhuang Y, Zhou M, Liu S, Wu J, Wang R, Chen C (2019). Effectiveness of personalized 3D printed models for patient education in degenerative lumbar disease. Patient Educ Couns.

[63] Meyer-Szary J, Luis MS, Mikulski S, Patel A, Schulz F, Tretiakow D, Fercho J, Jaguszewska K, Frankiewicz M, Pawłowska E, Targoński R, Szarpak ?, Dądela K, Sabiniewicz R, Kwiatkowska J (2022). The role of 3D printing in planning complex medical procedures and training of medical professionals-cross-sectional multispecialty review. Int J Environ Res Public Health.

[64] Baniasadi T, Ayyoubzadeh SM, Mohammadzadeh N (2020). Challenges and practical considerations in applying virtual reality in medical education and treatment. Oman Med J.

[65] Debarba HG, Montagud M, Chagué S, Herrero JG, Lacosta I, Langa SF, Charbonnier C (2022). Content format and quality of experience in virtual reality. Multimed Tools Appl.

[66] Marougkas A, Troussas C, Krouska A, Sgouropoulou C (2023). Virtual reality in education: a review of learning theories, approaches and methodologies for the last decade. Electronics.

[67] Arefin A, Khatri N, Kulkarni N, Egan P (2021). Polymer 3D printing review: materials, process, and design strategies for medical applications. Polymers (Basel).

[68] Tian Y, Chen C, Xu X, Wang J, Hou X, Li K, Lu X, Shi H, Lee E, Jiang H (2021). A review of 3D printing in dentistry: technologies, affecting factors, and applications. Scanning.

