# Supplementary material for: Perspectives of Medical Students and Developers Regarding Virtual Reality, Augmented Reality, Mixed Reality, and 3D Printing Technologies: Survey Study
Source: JMIR XR Spat Comput. 2024 May 7;1:e54230. doi: 10.2196/54230 (PMC13179110; doi:10.2196/54230)
Supplement: Multimedia Appendix 6 [file xr_v1i1e54230_app6.docx]

**Multimedia Appendix 6.** A survey on satisfaction with virtual reality, augmented reality, and 3D printing technologies.

| **Survey Questions** | | **5-point Likert Scale** | | | | |
| --- | --- | --- | --- | --- | --- | --- |
|  |  | Very Dissatisfied | Dissatisfied | Neutral | Satisfied | Very Satisfied |
| **Virtual Reality** | |  | | | | |
| Overall satisfaction | 1. Please select your overall satisfaction with VR^a^ technology. | 1 | 2 | 3 | 4 | 5 |
| Esthetics | 2. VR content allowed me to feel a sense of vividness. | 1 | 2 | 3 | 4 | 5 |
|  | 3. VR content is well-designed and harmonious overall. | 1 | 2 | 3 | 4 | 5 |
| Understanding of concept | 4. VR content was easy to understand by observing structures from desired angles. | 1 | 2 | 3 | 4 | 5 |
|  | 5. I was able to learn the structures provided in the content effectively. | 1 | 2 | 3 | 4 | 5 |
| Reality | 6. I felt like I was inside the provided content's space. | 1 | 2 | 3 | 4 | 5 |
|  | 7. The world within the content felt like it truly existed. | 1 | 2 | 3 | 4 | 5 |
| Spatial ability | 8. I gained a clear and intuitive understanding of spatial structures. | 1 | 2 | 3 | 4 | 5 |
|  | 9. I enhanced my understanding of spatial relationships of anatomical structures. | 1 | 2 | 3 | 4 | 5 |
| Immersion | 10. I wanted to continue the VR content practice and did not want to return to everyday life. | 1 | 2 | 3 | 4 | 5 |
|  | 11. I momentarily forgot about my daily life. | 1 | 2 | 3 | 4 | 5 |
| Continuous use intention | 12. I would like to repeat VR content practice in the future. | 1 | 2 | 3 | 4 | 5 |
|  | 13. I want to continue VR content practice consistently in the future. | 1 | 2 | 3 | 4 | 5 |
| Future use | 14. I believe VR technology will be used in clinical settings in the future. | 1 | 2 | 3 | 4 | 5 |
|  | 15. I believe VR technology will replace conventional methods in clinical settings in the future. | 1 | 2 | 3 | 4 | 5 |
| **Augmented Reality** | |  | | | | |
| Overall satisfaction | 1. Please select your overall satisfaction with AR^b^ technology. | 1 | 2 | 3 | 4 | 5 |
| Esthetics | 2. AR content allowed me to feel a sense of vividness. | 1 | 2 | 3 | 4 | 5 |
|  | 3. AR content is well-designed and harmonious overall. | 1 | 2 | 3 | 4 | 5 |
| Understanding of concept | 4. AR content was easy to understand by observing structures from desired angles. | 1 | 2 | 3 | 4 | 5 |
|  | 5. I was able to learn the structures provided in the content effectively. | 1 | 2 | 3 | 4 | 5 |
| Reality | 6. I felt like I was inside the provided content's space. | 1 | 2 | 3 | 4 | 5 |
|  | 7. The world within the content felt like it truly existed. | 1 | 2 | 3 | 4 | 5 |
| Spatial ability | 8. I gained a clear and intuitive understanding of spatial structures. | 1 | 2 | 3 | 4 | 5 |
|  | 9. I enhanced my understanding of spatial relationships of anatomical structures. | 1 | 2 | 3 | 4 | 5 |
| Immersion | 10. I wanted to continue the AR content practice and did not want to return to everyday life. | 1 | 2 | 3 | 4 | 5 |
|  | 11. I momentarily forgot about my daily life. | 1 | 2 | 3 | 4 | 5 |
| Continuous use intention | 12. I would like to repeat AR content practice in the future. | 1 | 2 | 3 | 4 | 5 |
|  | 13. I want to continue AR content practice consistently in the future. | 1 | 2 | 3 | 4 | 5 |
| Future use | 14. I believe AR technology will be used in clinical settings in the future. | 1 | 2 | 3 | 4 | 5 |
|  | 15. I believe AR technology will replace conventional methods in clinical settings in the future. | 1 | 2 | 3 | 4 | 5 |
| **3D Printing** | |  | | | | |
| Overall satisfaction | 16. Please select your overall satisfaction with 3DP^c^ technology. | 1 | 2 | 3 | 4 | 5 |
| Esthetics | 17. 3DP allowed me to feel a sense of vividness. | 1 | 2 | 3 | 4 | 5 |
|  | 18. 3DP is well-designed and harmonious overall. | 1 | 2 | 3 | 4 | 5 |
| Understanding of concept | 19. 3DP was easy to understand by observing structures from desired angles. | 1 | 2 | 3 | 4 | 5 |
|  | 20. I was able to learn the structures effectively. | 1 | 2 | 3 | 4 | 5 |
| Reality | 21. I felt like I was inside the 3D Printing's space. | 1 | 2 | 3 | 4 | 5 |
|  | 22. The world within 3DP felt like it truly existed. | 1 | 2 | 3 | 4 | 5 |
| Spatial ability | 23. I gained a clear and intuitive understanding of spatial structures. | 1 | 2 | 3 | 4 | 5 |
|  | 24. I enhanced my understanding of spatial relationships of anatomical structures. | 1 | 2 | 3 | 4 | 5 |
| Immersion | 25. I wanted to continue the 3DP practice and did not want to return to everyday life. | 1 | 2 | 3 | 4 | 5 |
|  | 26. I momentarily forgot about my daily life. | 1 | 2 | 3 | 4 | 5 |
| Continuous use intention | 27. I would like to repeat 3DP practice in the future. | 1 | 2 | 3 | 4 | 5 |
|  | 28. I want to continue 3DP practice consistently in the future. | 1 | 2 | 3 | 4 | 5 |
| Future use | 29. I believe 3DP technology will be used in clinical settings in the future. | 1 | 2 | 3 | 4 | 5 |
|  | 30. I believe 3DP technology will replace conventional methods in clinical settings in the future. | 1 | 2 | 3 | 4 | 5 |

^a^VR = virtual reality.

^b^AR = augmented reality.

^c^3DP = 3D printing.
