# Supplementary material for: Perspectives of Medical Students and Developers Regarding Virtual Reality, Augmented Reality, Mixed Reality, and 3D Printing Technologies: Survey Study
Source: JMIR XR Spat Comput. 2024 May 7;1:e54230. doi: 10.2196/54230 (PMC13179110; doi:10.2196/54230)
Supplement: Multimedia Appendix 7 [file xr_v1i1e54230_app7.docx]

**Multimedia Appendix 7.** The educational background of the software and content developers in this study.

1. The major of developers with Bachelor's degree (N=18).

- Electronic and Communication Engineering

- Game Engineering

- Nursing

- Food and Nutrition

- Telecommunication Engineering

- Game Programming

- Smart Mobile

- Allied Physics

- Military Information Engineering

- Electronic Communication

- Computer Science

- Control and Instrumentation Engineering

- Animation and Visual Effect

- Fine Art (N=2)

- Animation

- Cartoon Animation

- Environmental Sculpture

2. The major of developers with Master's degree (N=1).

- Software convergence

3. The major of developers with PhD in candidate (N=2).

- ICT Conversion Technology

- Mechanical Engineering and Science

4. The major of developers with PhD (N=4).

- Electronic and Telecommunication Engineering

- Computer Telecommunication Engineering

- Computational Biology

- Optoelectronics Display
