# Supplementary material for: Perspectives of Medical Students and Developers Regarding Virtual Reality, Augmented Reality, Mixed Reality, and 3D Printing Technologies: Survey Study
Source: JMIR XR Spat Comput. 2024 May 7;1:e54230. doi: 10.2196/54230 (PMC13179110; doi:10.2196/54230)
Supplement: Multimedia Appendix 8 [file xr_v1i1e54230_app8.docx]

**Multimedia Appendix 8.** Average satisfaction differences in virtual reality, augmented reality, and 3D printing between students and developers.

**Table S1.** Average satisfaction differences in virtual reality between students and developers (N=41).

| Variables | | | Satisfaction level | | | *t*(*P*) |
| --- | --- | --- | --- | --- | --- | --- |
|  |  |  | N | Mean | Std. Deviation |  |
| **Overall**  **satisfaction** | | Student | 15 | 4.53 | 0.64 | 5.38(<.001) |
|  |  | Developer | 26 | 3.31 | 0.74 |  |
| **Esthetics** | Vividness | Student | 15 | 4.40 | 0.74 | 4.04(<.001) |
|  |  | Developer | 26 | 3.46 | 0.71 |  |
|  | Design | Student | 15 | 4.40 | 0.72 | 4.01(<.001) |
|  |  | Developer | 26 | 3.38 | 0.80 |  |
| **Understanding**  **of concept** | Desired angles | Student | 15 | 4.47 | 0.74 | 3.68(<.001) |
|  |  | Developer | 26 | 3.62 | 0.70 |  |
|  | Comprehensive | Student | 15 | 4.33 | 1.11 | 2.67(.011) |
|  |  | Developer | 26 | 3.58 | 0.70 |  |
| **Reality** | Environment | Student | 15 | 4.40 | 0.74 | 2.85(.007) |
|  |  | Developer | 26 | 3.65 | 0.85 |  |
|  | Real world | Student | 15 | 4.00 | 1.00 | 2.84(.007) |
|  |  | Developer | 26 | 3.00 | 1.13 |  |
| **Spatial ability** | Intuitive understanding | Student | 15 | 4.33 | 0.98 | 2.90(.006) |
|  |  | Developer | 26 | 3.54 | 0.76 |  |
|  | Spatial perception | Student | 15 | 4.27 | 0.88 | 2.88(.006) |
|  |  | Developer | 26 | 3.58 | 0.64 |  |
| **Immersion** | Desire to continue | Student | 15 | 2.40 | 0.99 | 0.08(.94) |
|  |  | Developer | 26 | 2.42 | 0.86 |  |
|  | Forgetting about daily life | Student | 15 | 2.33 | 1.05 | 0.52(.60) |
|  |  | Developer | 26 | 2.50 | 0.95 |  |
| **Continuous use intension** | Repetition | Student | 15 | 3.93 | 1.03 | 2.11(.04) |
|  |  | Developer | 26 | 3.39 | 0.64 |  |
|  | Continuous use | Student | 15 | 4.00 | 1.00 | 2.41(.02) |
|  |  | Developer | 26 | 3.39 | 0.64 |  |
| **Future use** | Clinical field | Student | 15 | 4.27 | 0.88 | 3.15(.003) |
|  |  | Developer | 26 | 3.39 | 0.85 |  |
|  | Replacement | Student | 15 | 3.73 | 0.80 | 1.84(.07) |
|  |  | Developer | 26 | 3.23 | 0.86 |  |

**Table S2.** Average satisfaction differences in augmented reality between students and developers (N=41).

| Variables | | | Satisfaction level | | | *t*(*P*) |
| --- | --- | --- | --- | --- | --- | --- |
|  |  |  | N | Mean | Std. Deviation |  |
| **Overall**  **satisfaction** | | Student | 15 | 4.60 | 0.74 | 5.47(<.001) |
|  |  | Developer | 26 | 3.35 | 0.69 |  |
| **Esthetics** | Vividness | Student | 15 | 4.20 | 0.86 | 2.89(.006) |
|  |  | Developer | 26 | 3.42 | 0.81 |  |
|  | Design | Student | 15 | 4.47 | 0.64 | 4.83(<.001) |
|  |  | Developer | 26 | 3.27 | 0.83 |  |
| **Understanding**  **of concept** | Desired angles | Student | 15 | 3.93 | 1.16 | 1.40(.17) |
|  |  | Developer | 26 | 3.50 | 0.81 |  |
|  | Comprehensive | Student | 15 | 4.27 | 0.96 | 2.86(.007) |
|  |  | Developer | 26 | 3.46 | 0.81 |  |
| **Reality** | Environment | Student | 15 | 4.13 | 0.92 | 3.41(.002) |
|  |  | Developer | 26 | 3.08 | 0.98 |  |
|  | Real world | Student | 15 | 3.33 | 1.18 | 0.73(.47) |
|  |  | Developer | 26 | 3.08 | 1.02 |  |
| **Spatial ability** | Intuitive understanding | Student | 15 | 3.73 | 1.03 | 0.69(.49) |
|  |  | Developer | 26 | 3.54 | 0.76 |  |
|  | Spatial perception | Student | 15 | 3.93 | 0.96 | 1.66(.11) |
|  |  | Developer | 26 | 3.50 | 0.71 |  |
| **Immersion** | Desire to continue | Student | 15 | 2.13 | 0.64 | 1.91(.06) |
|  |  | Developer | 26 | 2.65 | 0.94 |  |
|  | Forgetting about daily life | Student | 15 | 2.33 | 0.82 | 0.94(.35) |
|  |  | Developer | 26 | 2.62 | 0.98 |  |
| **Continuous use intension** | Repetition | Student | 15 | 4.07 | 0.80 | 2.61(.01) |
|  |  | Developer | 26 | 3.50 | 0.58 |  |
|  | Continuous use | Student | 15 | 4.13 | 0.74 | 2.86(.007) |
|  |  | Developer | 26 | 3.50 | .65 |  |
| **Future use** | Clinical field | Student | 15 | 4.27 | .80 | 2.33(.03) |
|  |  | Developer | 26 | 3.61 | .90 |  |
|  | Replacement | Student | 15 | 4.07 | .88 | 2.47(.02) |
|  |  | Developer | 26 | 3.42 | .76 |  |

**Table S3.** Average satisfaction differences in 3D printing between students and developers (N=41).

| Variables | | | Satisfaction level | | | *t*(*P*) |
| --- | --- | --- | --- | --- | --- | --- |
|  |  |  | N | Mean | Std. Deviation |  |
| **Overall**  **satisfaction** | | Student | 15 | 4.40 | 0.63 | 3.29(.002) |
|  |  | Developer | 26 | 3.69 | 0.68 |  |
| **Esthetics** | Vividness | Student | 15 | 4.47 | 0.74 | 3.53(.001) |
|  |  | Developer | 26 | 3.65 | 0.69 |  |
|  | Design | Student | 15 | 4.60 | 0.74 | 3.33(.002) |
|  |  | Developer | 26 | 3.85 | 0.67 |  |
| **Understanding**  **of concept** | Desired angles | Student | 15 | 4.73 | 0.46 | 3.73(< .001) |
|  |  | Developer | 26 | 3.96 | 0.72 |  |
|  | Comprehensive | Student | 15 | 4.40 | 0.63 | 2.34(.02) |
|  |  | Developer | 26 | 3.92 | 0.63 |  |
| **Reality** | Environment | Student | 15 | 3.53 | 1.25 | 2.21(.03) |
|  |  | Developer | 26 | 2.77 | 0.95 |  |
|  | Real world | Student | 15 | 3.33 | 1.29 | 1.74(.09) |
|  |  | Developer | 26 | 2.73 | 0.92 |  |
| **Spatial ability** | Intuitive understanding | Student | 15 | 4.40 | 0.74 | 2.45(.02) |
|  |  | Developer | 26 | 3.85 | 0.67 |  |
|  | Spatial perception | Student | 15 | 4.33 | 0.72 | 2.43(.02) |
|  |  | Developer | 26 | 3.77 | 0.71 |  |
| **Immersion** | Desire to continue | Student | 15 | 2.40 | 0.83 | 0.08(.94) |
|  |  | Developer | 26 | 2.42 | 0.90 |  |
|  | Forgetting about daily life | Student | 15 | 2.40 | 0.51 | 0.34(.73) |
|  |  | Developer | 26 | 2.31 | 0.97 |  |
| **Continuous use intension** | Repetition | Student | 15 | 4.33 | 0.62 | 3.77(<.001) |
|  |  | Developer | 26 | 3.46 | 0.76 |  |
|  | Continuous use | Student | 15 | 4.20 | 0.68 | 2.93(.006) |
|  |  | Developer | 26 | 3.54 | 0.71 |  |
| **Future use** | Clinical field | Student | 15 | 4.33 | 0.72 | 2.06(.046) |
|  |  | Developer | 26 | 3.85 | 0.73 |  |
|  | Replacement | Student | 15 | 4.20 | 0.68 | 3.00(.005) |
|  |  | Developer | 26 | 3.42 | 0.86 |  |
