# Supplementary figures and images for: Perspectives of Medical Students and Developers Regarding Virtual Reality, Augmented Reality, Mixed Reality, and 3D Printing Technologies: Survey Study
Source: JMIR XR Spat Comput. 2024 May 7;1:e54230. doi: 10.2196/54230 (PMC13179110; doi:10.2196/54230)

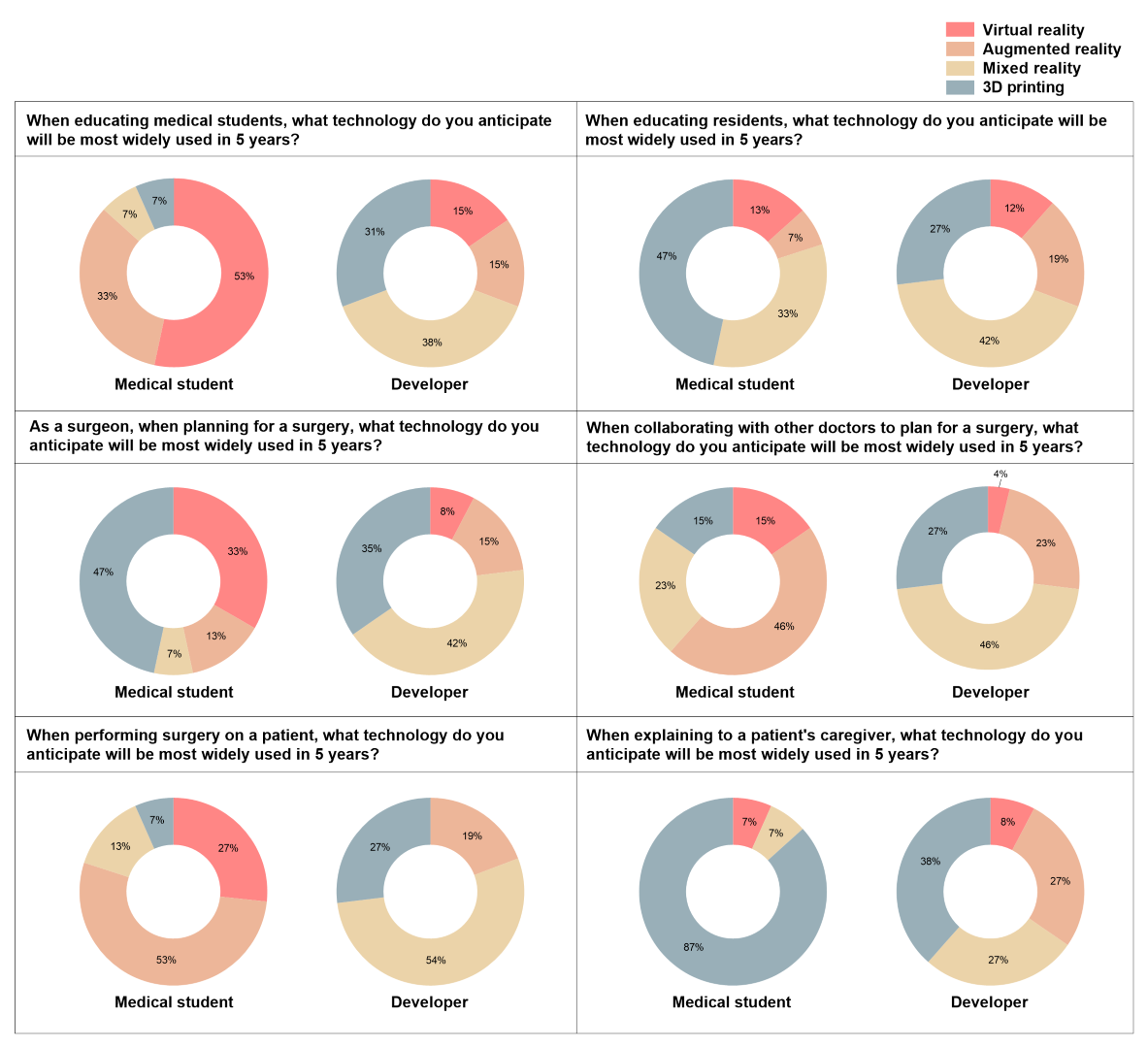

Supplement: Multimedia Appendix 9 [file xr_v1i1e54230_app9.png]

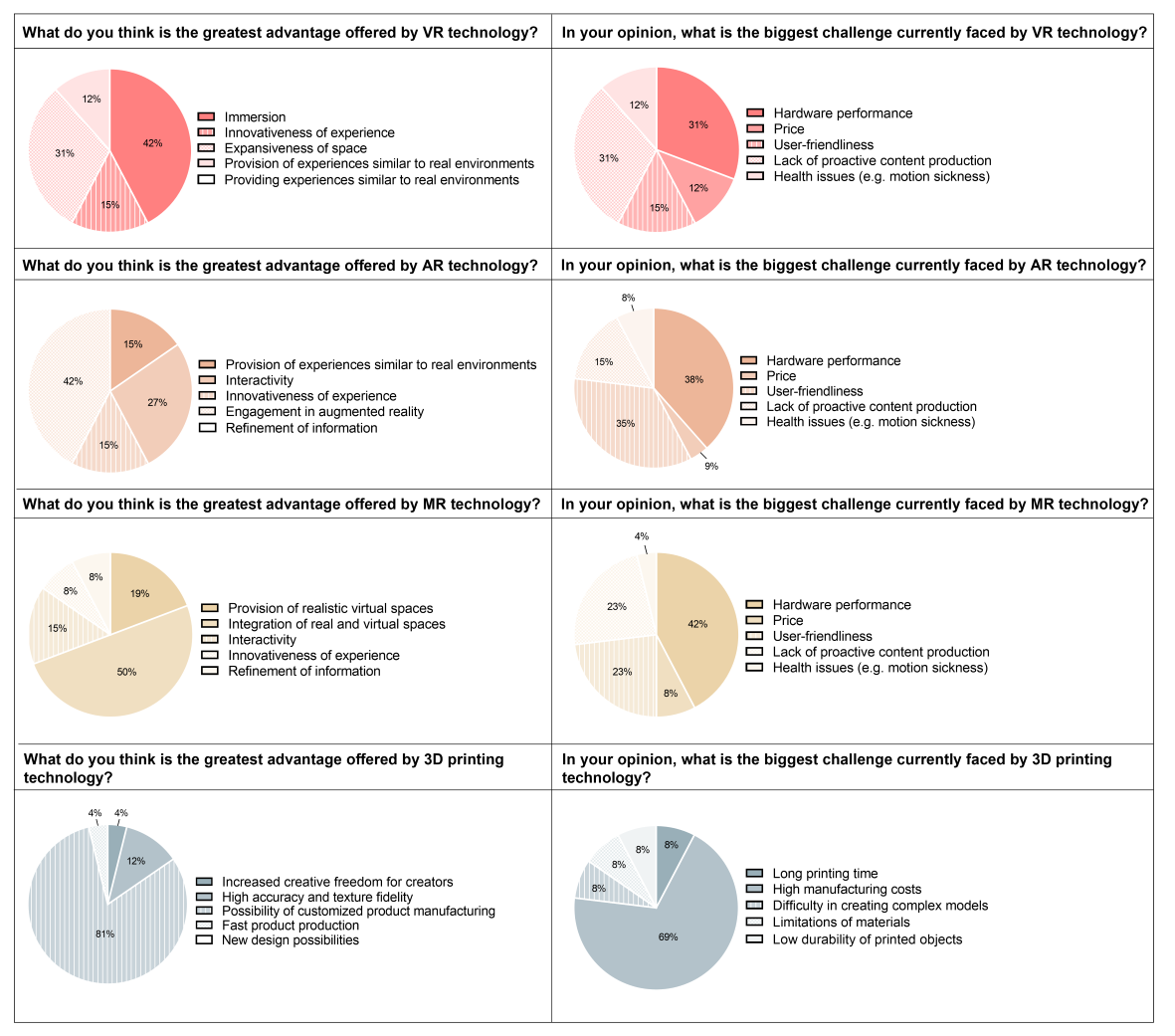

Supplement: Multimedia Appendix 10 [file xr_v1i1e54230_app10.png]
